# The Associations of Month of Birth With Body Mass Index, Waist Circumference, and Leg Length: Findings From the China Kadoorie Biobank of 0.5 Million Adults

**DOI:** 10.2188/jea.JE20140154

**Published:** 2015-03-05

**Authors:** Jun Lv, Canqing Yu, Yu Guo, Zheng Bian, Sarah Lewington, Huiyan Zhou, Yunlong Tan, Junshi Chen, Zhengming Chen, Liming Li

**Affiliations:** 1 Department of Epidemiology and Biostatistics, School of Public Health, Peking University Health Science Center, Beijing, China; 2 Chinese Academy of Medical Sciences, Beijing, China; 3 Clinical Trial Service Unit & Epidemiological Studies Unit (CTSU), Nuffield Department of Population Health, University of Oxford, Oxford, UK; 4 China National Center for Food Safety Risk Assessment, Beijing, China

**Keywords:** month of birth, BMI, waist circumference, leg length, vitamin D deficiency

## Abstract

**Background:**

Season of birth (SoB) has been linked with various health outcomes. This study aimed to examine the associations between month of birth (MoB) and adult measures of leg length (LL), body mass index (BMI), and waist circumference (WC).

**Methods:**

We analysed survey data from 10 geographically diverse areas of China obtained through the China Kadoorie Biobank. Analysis included 487 529 adults with BMI ≥ 18.5 kg/m^2^. A general linear model was used to examine the associations between MoB and adult measures of LL, BMI, and WC, adjusted for survey site, sex, age, education level, smoking habit, alcohol consumption, physical activity level, sedentary leisure time, height (only for WC and LL), and hip circumference (only for LL).

**Results:**

MoB was independently associated with both BMI and WC. Birth months in which participants had higher measures of adiposity were March–July for BMI and March–June for WC. The peak differences were 0.14 kg/m^2^ for BMI and 0.47 cm for WC. The association between MoB and LL depended on survey site. Participants who were born in February–August in four sites (Harbin, Henan, Gansu, and Hunan) had the shortest LL (all *P* < 0.01). The peak difference in mean LL was 0.21 cm. No statistically significant association between MoB and LL was noted in the other sites (Qingdao, Suzhou, Sichuan, Zhejiang, Liuzhou, and Haikou).

**Conclusions:**

These findings suggest that MoB is associated with variations in adult adiposity measures and LL among Chinese adults. Low exposure to ultraviolet B radiation and subsequent reduced levels of vitamin D during the late second and early third trimesters may be involved in these phenomena.

## INTRODUCTION

Obesity is a serious public health issue globally,^1^ and China is no exception.^2^^,^^3^ Besides recognition of established adult lifestyle behaviours and their environmental determinants,^1^ much attention is being focused on the developmental origins of adult obesity.^4^^,^^5^ There is increasing evidence that early life conditions, beginning with the intrauterine environment and continuing through the first few years of life, have long-term impacts on later health.^6^^,^^7^ Leg length (LL)—both in terms of absolute size and relative to total stature—has often been used as an anthropometric marker of the quality of the environment in early life in studies testing the hypothesis that early-life exposure to certain factors may have long-term impact on adult health.^8^^–^^10^ The general reasoning is that the vital organs of the head, thorax, and abdomen are protected from adversity at the expense of the less vital tissues of the limbs.^9^ LL has been linked to maternal smoking during pregnancy,^11^^,^^12^ birth weight,^11^ breastfeeding,^13^ energy intake at 4 years,^13^ and socioeconomic adversity in childhood.^11^ However, there remains considerable uncertainty about the adverse exposures during the prenatal and childhood periods, which may lead to shorter LL.

Season of birth (SoB) is a well-defined variable indicating various environmental factors in early life. These factors include not only meteorological factors and sunlight exposure, but also alterations in food supply and eating habits, energy expenditure (eg, outdoor physical activity and work load), air pollution, and exposure to infectious agents.^14^^–^^17^ If factors responsible for programming early in life change seasonally, then it stands to reason that individuals who are born in various seasons are influenced by those factors differently. Therefore, the SoB may be related to the later-life phenotype.^15^ To our knowledge, no study has examined the relationship between the SoB and LL. Only a few studies have examined the relationship between the SoB and adult adiposity in the population of Western countries, and their findings and explanations of underlying mechanism were mixed.^18^^–^^22^ No study has reported on the association between SoB and adult adiposity in the Chinese population.

Here, we analysed the baseline survey data from the China Kadoorie Biobank (CKB) of 0.5 million adults recruited from 10 geographically diverse areas of China.^23^^,^^24^ The main aim of the current study was to examine the associations between the month of birth (MoB) and adult measures of LL and general (body mass index [BMI]) and central adiposity (waist circumference [WC]).

## METHODS

### Study design and participants

Details of the CKB study design and characteristics of the study participants have been described elsewhere.^23^^,^^24^ Briefly, 512 891 participants aged 30–79 years were recruited between 2004 and 2008 from 5 urban and 5 rural areas of China (see eTable 1 for geographic coordinates and climate characteristics of the 10 survey sites and eFigure 1 for locations of the 10 survey sites). Selection of the survey sites was based on local patterns of disease and exposure to certain risk factors, population stability, quality of death and disease registries, and local commitment and capacity. Within each area, permanent residents without major disabilities in each of the 100–150 administrative units (rural villages or urban residential committees) that were selected for the study were identified from local records and sent a letter and leaflet inviting them to participate. The participation rate was 33% in rural areas and 27% in urban areas, and the main reasons for non-participation (reported anecdotally by field staff) were absence from the home and reluctance to spend time visiting the screening centre.

Ethical approval for the CKB study was obtained from the Ethics Review Committee of the Chinese Center for Disease Control and Prevention (Beijing, China) and the Oxford Tropical Research Ethics Committee, University of Oxford (UK). In addition, approvals were issued by the institutional research boards at the local Center for Disease Control and Prevention at each of the 10 survey sites. Finally, written informed consent was obtained from participants.

### Measures and variables

At the baseline survey, trained interviewers administered a standardised questionnaire using a laptop-based direct data-entry system, with built-in functions to prevent logical errors and missing items. The questionnaire included detailed questions on socio-demographic status, medical history and health status, smoking habit, alcohol consumption, diet, physical activity, and other lifestyle behaviours. The physical measurement procedures were standardised across the 10 survey sites, and all physical measurements were conducted by trained staff using a standard protocol and instruments. All of the utilised devices were regularly maintained and calibrated to ensure consistency in measurements.

The predictor variable of interest in this study was MoB. The outcome variables were BMI, WC, and LL. The participants did not wear shoes during their height and weight measurement. Standing height and sitting height (length of the body from buttocks to the crown of the head) were measured to the nearest 0.1 cm using a manufactured instrument. LL was calculated as the difference between standing and sitting height. Weight was measured to the nearest 0.1 kg using a TANITA TBF-300GS body composition analyser (Tanita Corp., Tokyo, Japan). BMI was calculated as weight in kilograms divided by the square of the standing height in metres. WC was measured midway between the iliac crest and the lower rib margin at the end of normal expiration, and hip circumference (HC) was measured at the widest level over the greater trochanters using a plastic flexible tape to the nearest 0.1 cm.

Other covariates included survey site, sex, age, the highest education completed (no formal school, primary school, middle school, high school, or college/university), smoking habit (never, occasional, former, or current regular), alcohol consumption (never, occasional, former, or current regular),^25^ total physical activity in metabolic equivalent hours per day (MET-hours/day),^26^ and sedentary leisure time (hours/week).^26^

### Statistical analyses

The current analysis included participants who had a weight between 30 kg and 160 kg, a height between 145 cm and 200 cm for males or between 140 cm and 200 cm for females, a BMI between 18.5 kg/m^2^ and 45.0 kg/m^2^, and a WC between 50 cm and 150 cm. A total of 487 529 participants remained in this analysis. A general linear model was used to test for differences in mean BMI, WC, and LL between 12 MoB groups, adjusting for survey site, sex, age, education, smoking habit, alcohol consumption, amount of physical activity, and sedentary leisure time. Analyses using WC or LL as outcome measures were additionally adjusted for height. Further, considering that a thicker gluteo-femoral fat thickness will increase sitting height and artificially decrease LL,^27^ we additionally adjusted for HC in the analysis of LL. Bonferroni’s method was used to adjust the *P*-values for multiple comparisons. The presence of interactions of MoB with sex and survey site was also tested. All of the statistical analyses were performed using Stata version 13.1 (StataCorp LP., College Station, TX, USA).^28^

## RESULTS

Overall, of the 487 529 participants included in the analysis, 200 529 (41.1%) were men, and 268 962 (55.2%) resided in rural areas. At the time of the survey, the mean age was 51.3 ± 10.6 years. More men (58.7%) than women (44.1%) finished middle school or above. Both the prevalence of current smoking (60.6% compared with 2.2%) and of drinking (33.7% compared with 2.0%) were higher among men than women. Men were also less physically active (25.3 compared with 26.8 MET-hours/day) and spent more time on leisure-time sedentary behaviors (21.8 compared with 20.8 hours/week) than women. Women had slightly higher BMI (24.1 compared with 23.7 kg/m^2^) and larger HC (91.6 compared with 91.1 cm) but lower WC (79.8 compared with 82.7 cm) and shorter LL (71.0 compared with 76.9 cm) than men (Table 1).

**Table 1.** Main characteristics of study participants by sex

	Men (*n* = 200 529)	Women (*n* = 287 000)	*P* for gender difference
Age, years (mean ± SD)	52.1 ± 10.8	50.7 ± 10.3	<0.001
Education (%)			<0.001
Illiterate	8.5	24.7	
Elementary	32.8	31.2	
Middle school	32.8	25.8	
High school	17.8	13.8	
College and above	8.1	4.5	
Tobacco use (%)			<0.001
Nonsmoker	14.5	95.2	
Occasional smoker	11.4	1.8	
Former smoker	13.4	0.8	
Regular smoker	60.6	2.2	
Alcohol use (%)			<0.001
Nondrinker	19.7	63.0	
Occasional drinker	38.0	34.1	
Former drinker	8.7	0.9	
Regular drinker	33.7	2.0	
Physical activity, MET-hours/day^a^ (mean ± SD)	25.3 ± 12.0	26.8 ± 10.3	<0.001
Sedentary leisure time, hours/week^a^ (mean ± SD)	21.8 ± 10.7	20.8 ± 10.9	<0.001
Height, cm^a^ (mean ± SD)	165.3 ± 6.4	154.4 ± 5.7	<0.001
LL, cm^a^ (mean ± SD)	76.9 ± 4.0	71.0 ± 3.6	<0.001
Weight, kg^a^ (mean ± SD)	65.0 ± 10.4	57.5 ± 8.9	<0.001
BMI, kg/m^2 a^ (mean ± SD)	23.7 ± 3.1	24.1 ± 3.2	<0.001
WC, cm^a^ (mean ± SD)	82.7 ± 9.4	79.8 ± 9.1	<0.001
HC, cm^a^ (mean ± SD)	91.1 ± 6.6	91.6 ± 6.6	<0.001

MET-hours/day, metabolic equivalent hours per day; LL, leg length; BMI, body mass index; WC, waist circumference; HC, hip circumference.

^a^*P*-values for gender difference were adjusted for age and survey site.

**Figure 1.**
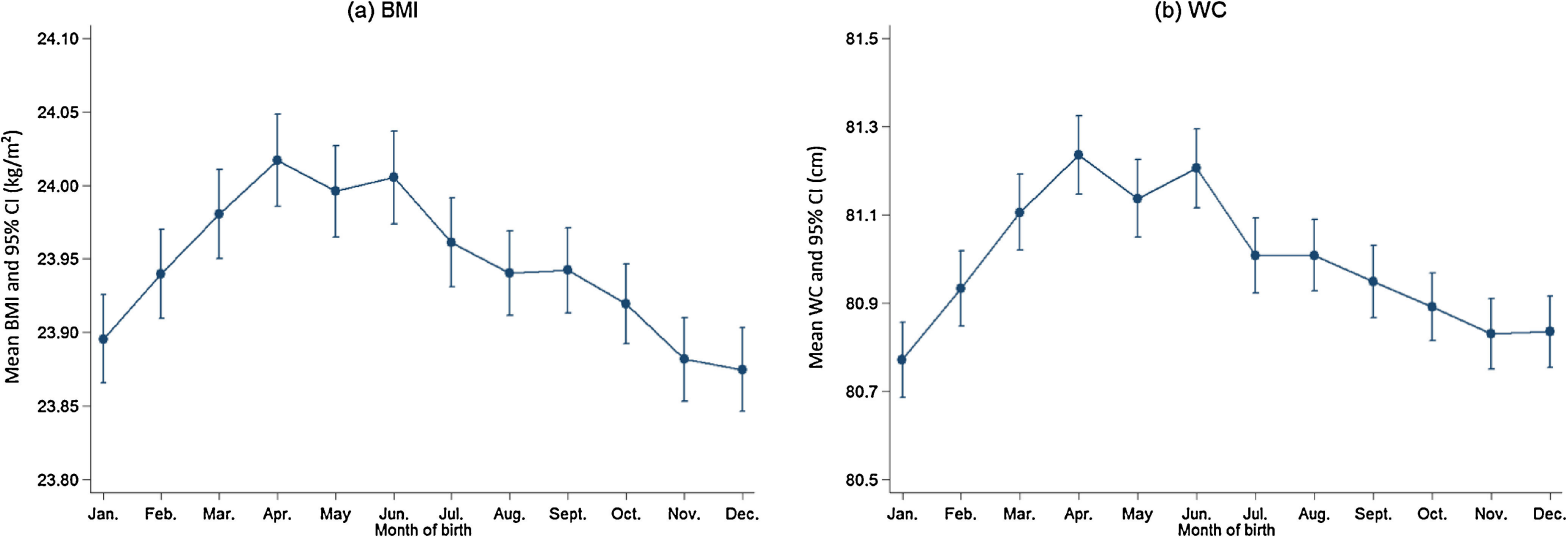
Adjusted means of BMI and WC with 95% CI by month of birth among study participants

MoB was independently associated with both BMI (*F* = 9.70, *P* < 0.001) and WC (*F* = 12.05, *P* < 0.001) after accounting for survey site, sex, age, education, smoking, alcohol use, physical activity, sedentary leisure time, and height (adjusting only for WC). Associations of MoB with BMI and WC were independent of gender (*P* for interaction = 0.725 for BMI and 0.459 for WC) and survey site (*P* for interaction = 0.080 for BMI and 0.115 for WC). Adjusted mean BMI and WC by MoB are presented in Table 2 and Figure 1. After correcting for multiple comparisons, participants who were born in March–July had higher BMI than those who were born in other months. The maximum difference in mean BMI across twelve months was 0.14 kg/m^2^ (maximum: April, 24.02 kg/m^2^; minimum: December, 23.87 kg/m^2^). A similar trend was also noted for WC. Participants who were born in March–June had larger WC than those who were born in other months. The maximum difference in mean WC across 12 months was 0.47 cm (maximum: April, 81.24 cm; minimum: January, 80.77 cm). Adjusted prevalence of general and central adiposity by MoB presented similar trends (Table 2).

**Table 2.** Means of BMI and WC and prevalence of general and central adiposity by MoB among study participants

MoB	*n*	BMI (kg/m^2^)									WC (cm)							
	
		24.0 to <27.9 (%)^a^	≥28.0 (%)^a^	Mean^a^	(SE)	Bonferroni-corrected multiple comparison^b^					Men ≥85 Women ≥80 (%)^a^	Mean^a^	(SE)	Bonferroni-corrected multiple comparison^b^				
Jan.	38 987	34.0	10.9	23.90	(0.02)	A	B				43.1	80.77	(0.04)	A				
Feb.	38 850	34.8	11.0	23.94	(0.02)	A	B	C	D		43.7	80.93	(0.04)	A	B	C		
Mar.	38 743	35.3	11.2	23.98	(0.02)			C	D	E	44.5	81.11	(0.04)			C	D	E
Apr.	36 424	35.1	11.7	24.02	(0.02)					E	45.2	81.24	(0.05)					E
May	36 677	34.8	11.6	24.00	(0.02)				D	E	44.5	81.14	(0.05)			C	D	E
Jun.	35 638	35.2	11.5	24.01	(0.02)				D	E	45.0	81.21	(0.05)				D	E
Jul.	39 504	34.8	11.3	23.96	(0.02)		B	C	D	E	44.1	81.01	(0.04)		B	C	D	
Aug.	43 111	34.7	11.1	23.94	(0.01)	A	B	C	D		43.9	81.01	(0.04)		B	C	D	
Sept.	42 780	34.6	11.0	23.94	(0.01)	A	B	C	D		43.6	80.95	(0.04)	A	B	C		
Oct.	48 669	34.7	10.9	23.92	(0.01)	A	B	C			43.6	80.89	(0.04)	A	B			
Nov.	44 086	34.6	10.5	23.88	(0.01)	A					43.3	80.83	(0.04)	A	B			
Dec.	44 060	34.4	10.6	23.87	(0.01)	A					43.5	80.83	(0.04)	A	B			

BMI, body mass index; WC, waist circumference; MoB, month of birth; SE, standard error.

^a^Prevalences and means adjusted for survey sites, sex, age, education, smoking, alcohol use, physical activity, sedentary leisure time, and height (for WC only).

^b^Means sharing the same letter were not significantly different at Bonferroni-corrected level of significance.

**Figure 2.**
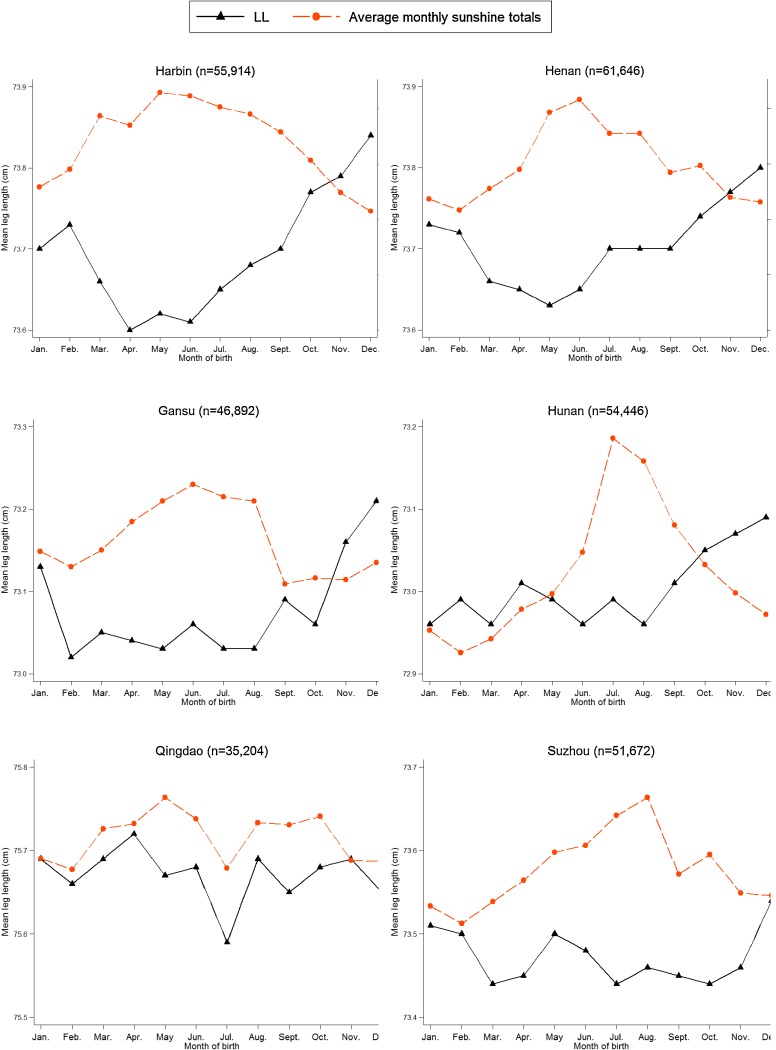

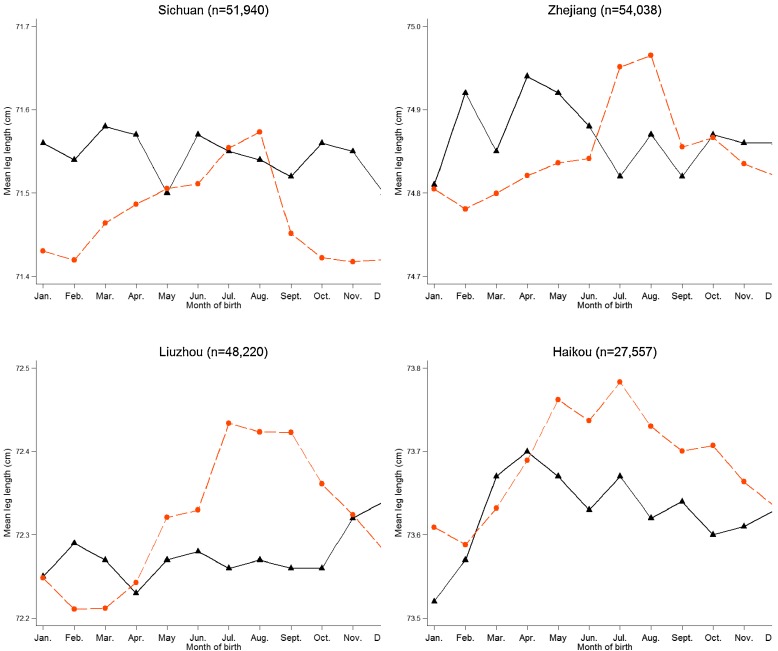
Monthly variation in mean leg length (cm) by survey site, with average monthly sunshine totals as reference. The sunshine data were collected by the weather station nearest to each survey site from 1951 to 1980, which were made available through the Database for Climate Resources (http://www.data.ac.cn). The average monthly sunshine total was used to measure the duration of sunshine in a month and was expressed as an average of 30 years from 1951 to 1980.

**Figure 3.**
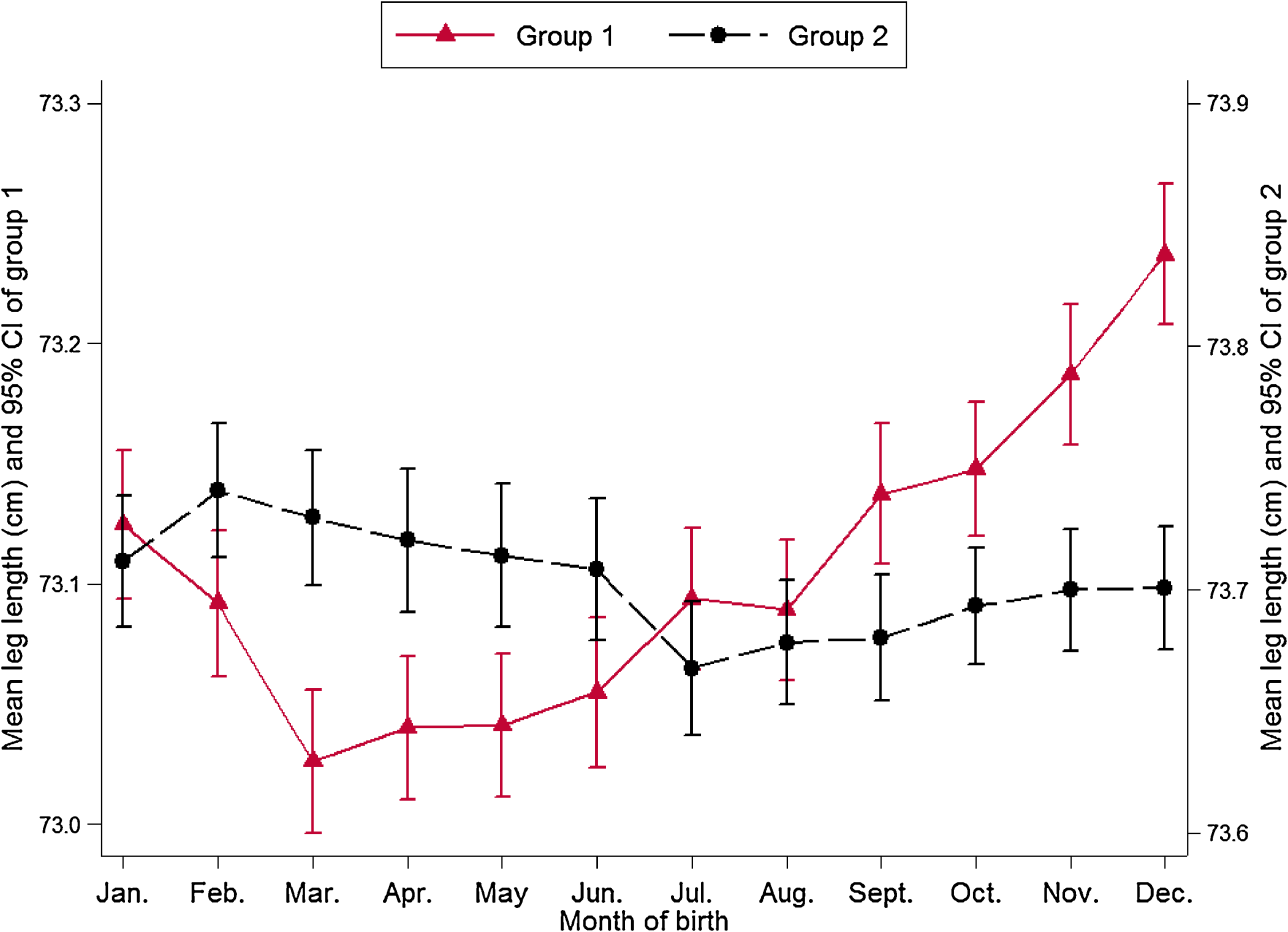
Mean leg length (cm) with 95% confidence intervals by month of birth among 218 898 participants of group 1 and 268 631 participants of group 2

Association of MoB with LL was independent of gender (*P* for interaction = 0.854) but dependent on survey site (*P* for interaction < 0.001), after adjusting for survey site, sex, age, education level, smoking habit, alcohol consumption, physical activity amount, sedentary leisure time, height, and HC. MoB was independently associated with LL among participants in Harbin (*P* < 0.001), Henan (*P* < 0.001), Gansu (*P* < 0.001), and Hunan (*P* = 0.006) (Figure 2). These four survey sites showed similar seasonal variations in LL; participants who were born at the end of the year had longer LL. No statistically significant association between MoB and LL was detected in the other six survey sites. The means of LL by MoB, stratified by whether or not the survey sites showed seasonal variation in LL, are presented in Table 3 and Figure 3. Group 1, including participants from Harbin, Henan, Gansu, and Hunan, showed statistically significant variation in LL associated with MoB. Participants who were born in February–August had shorter LL, and those who were born in November–December had longer LL. The maximum difference in mean LL across twelve months was 0.21 cm (maximum: December, 73.24 cm; minimum: March, 73.03 cm). No statistically significant association between MoB and LL was found in group 2, which included participants from Qingdao, Suzhou, Sichuan, Zhejiang, Liuzhou, and Haikou.

**Table 3.** Mean leg length of two groups of survey sites^a^ by MoB among study participants

MoB	Group 1 (*n* = 218 898)							Group 2 (*n* = 268 631)		
	
	Mean LL, cm^b^	(SE)	Bonferroni-corrected multiple comparison^c^					Mean LL, cm^b^	(SE)	Bonferroni-corrected multiple comparison^c^
Jan.	73.12	(0.02)		B	C	D		73.71	(0.01)	F
Feb.	73.09	(0.02)	A	B	C			73.74	(0.01)	F
Mar.	73.03	(0.02)	A					73.73	(0.01)	F
Apr.	73.04	(0.02)	A					73.72	(0.02)	F
May	73.04	(0.02)	A					73.71	(0.01)	F
Jun.	73.05	(0.02)	A	B				73.71	(0.01)	F
Jul.	73.09	(0.02)	A	B	C			73.67	(0.01)	F
Aug.	73.09	(0.01)	A	B	C			73.68	(0.01)	F
Sept.	73.14	(0.01)			C	D		73.68	(0.01)	F
Oct.	73.15	(0.01)			C	D		73.69	(0.01)	F
Nov.	73.19	(0.01)				D	E	73.70	(0.01)	F
Dec.	73.24	(0.01)					E	73.70	(0.01)	F

LL, leg length; MoB, month of birth; SE, standard error.

^a^Group 1 including Harbin, Henan, Gansu, and Hunan; group 2 including Qingdao, Suzhou, Sichuan, Zhejiang, Liuzhou, and Haikou.

^b^Means LL adjusted for survey sites, sex, age, education, smoking, alcohol use, physical activity, sedentary leisure time, height, and HC.

^c^Means sharing the same letter were not significantly different at Bonferroni-corrected level of significance.

## DISCUSSION

The present study is the largest population-based study ever to report on the associations of MoB with adult adiposity and LL measures. The study’s findings suggest that spring- and early summer-born adults had higher BMI and WC and shorter LL than autumn- and winter-born adults in China. Participants at all 10 survey sites showed seasonal variations in BMI and WC, but only 4 sites showed seasonal variation in LL. The associations between MoB and adult measures of adiposity and LL were independent of gender.

Few studies have examined the association between SoB and adult adiposity. Inconsistent findings and mixed explanations of underlying mechanism have been reported, including speculation that early temperature exposure is associated with physiological regulation of birth weight or that extremely low ambient temperature influences the conceived embryo or the selection of the sperm that fuses with the ovum.^19^^–^^21^ However, the findings from the current study appeared to more strongly suggest the possible etiological role of prenatal maternal exposure to ultraviolet B radiation (UVB) and vitamin D status in adult chronic diseases.^29^^–^^31^

Sun exposure provides one of the major ways to meet vitamin D requirements during pregnancy, especially for those of older generations. For latitudes with distinct seasonal changes in availability of UVB, there is a well-documented seasonal cycle in vitamin D status, with a maximum in late summer and a minimum in late winter.^32^ Low vitamin D during gestation may strongly influence susceptibility to obesity later in life.^33^

**Figure 4.**
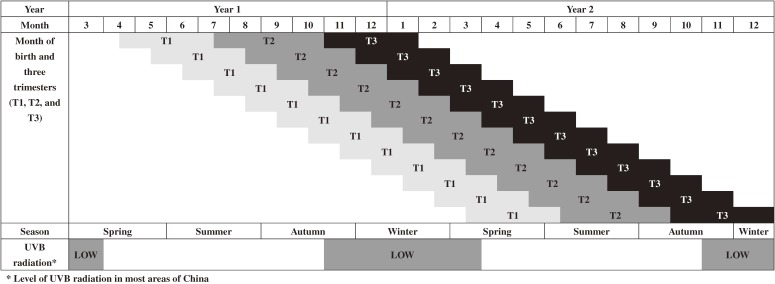
Month of birth and three trimesters in relation to the calendar month of year, season, and the level of UVB radiation

However, relatively little is known about the critical period of fetal growth at which low vitamin D is of particular importance. Low vitamin D during the second or third trimester of pregnancy has been suggested to be associated with adult obesity.^31^^,^^33^ In the current study, spring and early summer peaks and winter troughs for BMI and WC were evident. Mothers with babies born in spring and early summer had low sun-related UVB exposure in winter, and hence, lower vitamin D exposure for their offspring during the late second and early third trimesters (Figure 4). Babies who were born in winter had high UVB exposure during the late second and early third trimesters, which occurred in summer and early autumn. Up to now, a few studies have examined the association between lower maternal vitamin D levels in later pregnancy and offspring adiposity at age 6 years^34^; at birth, age 9 months and age 9 years^35^; at ages 5 and 9.5 years^36^; and at age 35 years,^37^ and shown inconsistent results. Further studies are warranted to test the association between prenatal vitamin D status and adult adiposity in an adult population older than 35 years.

To our knowledge, no previous study has examined the relationship between prenatal vitamin D status and LL. LL in the present study followed an opposite seasonal trend to that of adult adiposity measures, for which maternal UVB exposure (and hence vitamin D status) is still a reasonable explanation. For spring- and summer-born babies, shorter LL, together with higher adiposity measures, could be the adverse outcome of lower UVB exposure in winter during their second and early third trimesters.

However, this association between MoB and LL was only observed in four survey sites. Three of them, including Harbin, Henan, and Gansu, have higher latitudes. Lucas et al found that seasonal variation in vitamin D status in Australian adults (living in an area spanning 27°–43° South) had greater amplitude compared to Australian adults living in a different region, and vitamin D deficiency increased with increasing latitude, reflecting stronger seasonal variation in ambient UVB and less skin exposure due to colder temperatures.^38^ Hunan, being another site presenting seasonal variation in LL, has lower latitude than seven other study sites but the greatest yearly sunshine variation of all the study sites (see eTable 1). Besides significant seasonal variation in sunshine, peak and trough windows are also supposed to last long enough to influence vitamin D status. These might explain why null results were found for the other six survey sites. Results in the present study imply that prenatal vitamin D status might be a potential pathway linking LL with adult risks of obesity^39^^,^^40^ and a range of chronic diseases.^8^^–^^10^ However, seasonal variation in LL depending on region suggests that LL can only be used as anthropometric biomarker of prenatal vitamin D status in some geographic regions.

The large sample size of the current study ensured sufficient statistical power to identify a relatively small difference. The study participants, who resided across a range of latitudes, provided a sound basis for observing that individuals living in low-latitude and lower- to middle-latitude regions of China also had seasonal low UVB exposure, and hence, higher measures of adult adiposity. Although the current study is unable to precisely determine the reason for MoB variation in adult measures of LL, general adiposity, and central adiposity, the study improves our current understanding of a potential association of prenatal low exposure to vitamin D during late second and early third trimesters with short LL and adiposity in later life. Vitamin D insufficiency during pregnancy is highly prevalent in a diverse range of populations living at various latitudes and in both developed and developing societies.^29^^,^^33^ The adverse effects of vitamin D insufficiency on offspring during development and later in life have also been extended greatly.^29^^–^^31^ From a public health perspective, the potential to prevent common chronic diseases via low-cost, simple, and safe food fortification is an attractive option.^33^

Some potential limitations of this study warrant consideration. First, we used MoB in the analysis and then speculated the period of trimester during which the mothers may have had low UVB exposure without considering preterm birth. Second, participants were geographically grouped based on their residence at the time of the survey rather than their place of birth. Given that these survey sites were not located in old, well-developed, large cities, it is unlikely that many local residents, especially those of older residents, were migrants from distant locations across the provinces. Third, we adjusted the analyses for socioeconomic and lifestyle variables at the time of the survey, but we failed to include additional potential confounders in early life, such as mother’s socio-demographic characteristics, health conditions, and lifestyle habits during pregnancy; adverse birth outcomes; postnatal feeding; and child growth and development, potentially biasing the findings. A lack of documented prenatal, birth, and child health records for a vast majority of Chinese citizens is the main reason for this missing information. Fourth, Bogin et al suggested that there is a significant bias when LL are estimated from stature and sitting height in populations with a high percentage of overweight and obesity.^27^ Lower extremity length should be measured directly. Although China’s obesity problem is increasing rapidly, the overall obesity rate is still relatively low. In addition, HC was included in the model to adjust gluteo-femoral fat thickness, mitigating any bias.

In summary, the present study provides the largest population evidence to date that MoB is associated with variations in adult measures of both general and central adiposity among Chinese adults. The association between MoB and LL was only seen in areas with possibly greater variation in seasonal UVB exposure. On average, individuals who were born in spring and early summer had higher BMI and WC and shorter LL than those who were born in autumn and winter. This is also the first-ever population-based evidence linking low prenatal sun-related UVB exposure (and lower vitamin D by proxy) to LL and the susceptibility to obesity later in life and also suggesting that late second and early third trimesters might be a critical period for the above-mentioned effects Although such evidence is non-specific, it warrants further studies in this area. Long-term follow-up data from this CKB study may provide prospective evidence for possible associations between MoB and many later health outcomes.

## ONLINE ONLY MATERIALS

eTable 1. Geographic coordinates and climate characteristics based on 30-year period from 1951 to 1980 for 10 survey sites.

eFigure 1. Locations of the 10 survey sites.
