# Supplementary material for: The Associations of Month of Birth With Body Mass Index, Waist Circumference, and Leg Length: Findings From the China Kadoorie Biobank of 0.5 Million Adults
Source: J Epidemiol. 2015 Mar 5;25(3):221–30. doi: 10.2188/jea.JE20140154 (PMC4340999; doi:10.2188/jea.JE20140154)
Supplement: eTable 1. [file je-25-221-s001.pdf]

eTable 1. Geographic coordinates and climate characteristics* based on 30-year period from 1951 to 1980 for 10 survey sites

Province	Survey sites	Residence	Latitude (°N)	Longitude (°E)	Average annual sunshine (hours)	Average annual sunshine range (hours)	Average annual temperature (°C)	Average annual temperature range (°C)
Heilongjiang	Harbin	Urban	45.75	126.63	2,641.1	107.3	3.6	42.2
Shandong	Qingdao	Urban	36.08	120.33	2,497.5	63.1	12.2	26.3
Henan	Huixian	Rural	35.46	113.80	2,385.2	100.0	14.2	27.6
Gansu	Tianshui	Rural	34.58	105.73	2,032.2	88.8	10.7	25.4
Jiangsu	Suzhou	Urban	31.30	120.60	2,155.0	110.7	15.3	26.0
Sichuan	Pengzhou	Rural	30.98	103.93	1,228.3	114.0	16.2	20.1
Zhejiang	Tongxiang	Rural	30.63	120.53	1,903.8	134.9	16.2	24.8
Hunan	Liuyang	Rural	28.14	113.63	1,677.2	191.1	17.2	24.6
Guangxi	Liuzhou	Urban	24.32	109.38	1,635.0	163.6	20.5	18.5
Hainan	Haikou	Urban	20.04	110.34	2,239.8	143.0	23.8	11.2

*The data for the two elements of climate (temperature and sunshine) were collected by the weather station nearest each survey site from 1951 to 1980. These data were made available through the Database for Climate Resources (http://www.data.ac.cn), which is maintained by the Institute of Geographic Sciences and Natural Resources Research (IGSNRR) of the Chinese Academy of Sciences (CAS). The average annual sunshine was used to measure the duration of sunshine in a year and was expressed as an average of 30 years from 1951 to 1980. The average annual ranges of sunshine and temperature were the difference between the highest and lowest average monthly values from 1951 to 1980.
