# Supplementary figures and images for: The Associations of Month of Birth With Body Mass Index, Waist Circumference, and Leg Length: Findings From the China Kadoorie Biobank of 0.5 Million Adults
Source: J Epidemiol. 2015 Mar 5;25(3):221–30. doi: 10.2188/jea.JE20140154 (PMC4340999; doi:10.2188/jea.JE20140154)

**eFigure 1. Locations of the 10 survey sites**

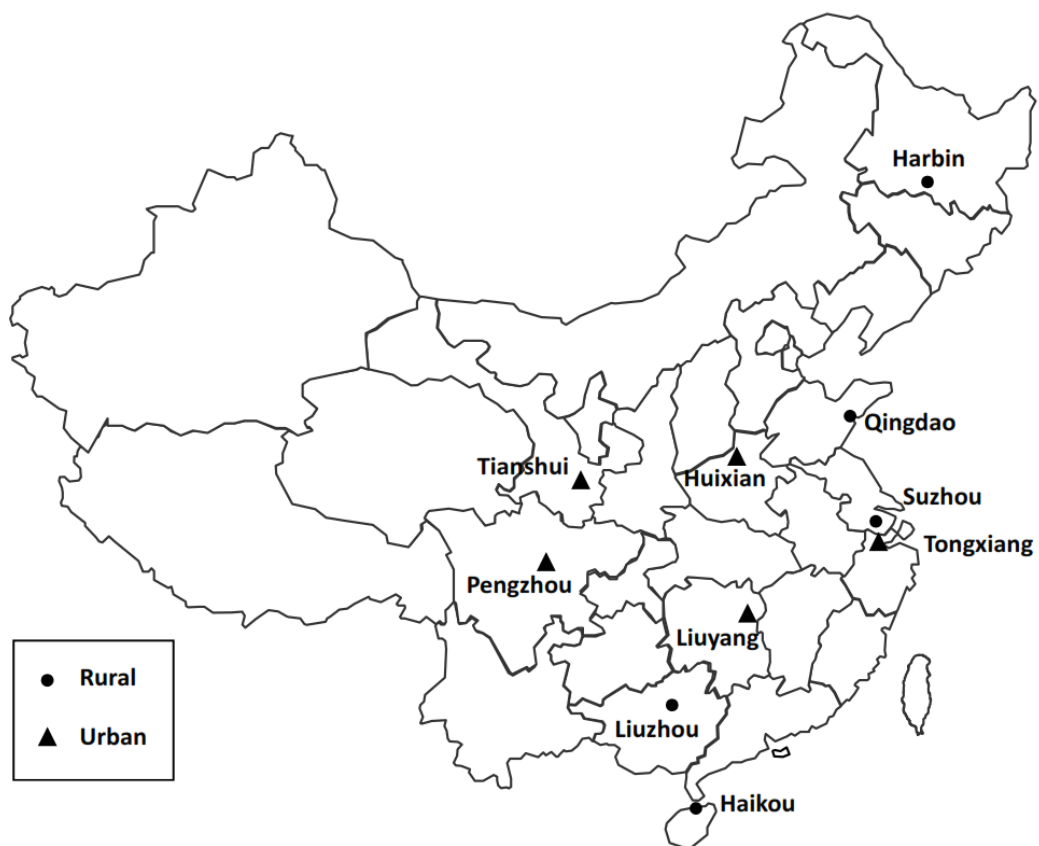

Supplement: eFigure 1. [file je-25-221-s002.pdf]
